# Making Hands Jive: How the Body Manages Hand Coordination

**DOI:** 10.1371/journal.pbio.0040196

**Published:** 2006-05-09

**Authors:** Mason Inman

Unscrewing the lid of a honey jar stuck tight takes more than simply muscle power and a vice-like grip. It also takes coordination between your hands—one twisting the lid and the other holding the jar—to open the jar without it spinning out of your grasp.

But you might not suspect that in tasks like this, you unconsciously appoint one of your hands the leader and assign the other a supporting role—and the leader is not necessarily your writing hand. A new study reveals that people are surprisingly flexible in assigning these roles, and that they can quickly switch dominance between hands depending on the nature of the task. This dominance turns out to be thoroughgoing, from the brain's cortex down to specific muscles in each hand.

In the new experiments, Roland S. Johansson and colleagues had 37 people, all right-handers, play a video game similar to the arcade classic Whac-A-Mole: when a target appeared in a random spot on a video screen, the players moved a cursor to touch the target as quickly as possible. Key to the experiments was a custom-made controller, a rectangular box with knobs on two ends, that the players used. To move the cursor horizontally on the screen, the players pushed the controller's knobs inward or pulled them out; twisting the knobs, like screwing or unscrewing a jar's lid, sent the cursor up or down.

With this setup, the researchers could see whether a person's hands were acting perfectly in concert or whether one hand took a dominant role, acting a bit before the other or pushing or twisting harder than the other. The researchers found that one hand did consistently dominate, so the body of the controller would shift or twist slightly at first, before the supporting hand had a chance to compensate.

But which hand was dominant depended on the rules of the game, and the players were flexible, quickly switching dominance between their hands when the rules of the game changed. For example, when the game was set up so squeezing the knobs inward moved the cursor to the left, then the players would make their right hand dominant. Conversely, when the researchers changed the rules so an inward squeeze sent the cursor right, then the left hand was dominant.

Johansson and colleagues found that the dominance of one hand manifests differently in each half of the body depending on the rules of the game. In the dominant hand, the muscles were stiffer and contracted at a higher rate, whereas the supporting hand was more relaxed and could compensate for the forces from the dominant hand. Also, functional magnetic resonance imaging (fMRI) showed that in motor-control areas of the brain's cortex and cerebellum, the center of activity switched between the brain's hemispheres when the rules of the game changed. Finally, the researchers used transcranial magnetic stimulation (TMS), bursts of magnetic fields that can boost activity in a specific brain region, to study how the brain's signals reached the hands. They found that the corticospinal pathways, which connect the brain's primary motor cortices with the spinal cord, were especially active only on the side controlling the dominant hand.

Together these findings suggest the body has a flexible system for assigning dominance to one hand while suppressing activity for the other hand so it can assume a supporting role, the authors argue. Dominance could come from the top down, from goals processed in the higher levels in the brain, or from the bottom up, perhaps from tactile sense of objects—or from a combination of the two. What's clear, however, is that the hands don't have to fight it out.

## 

**Figure:**
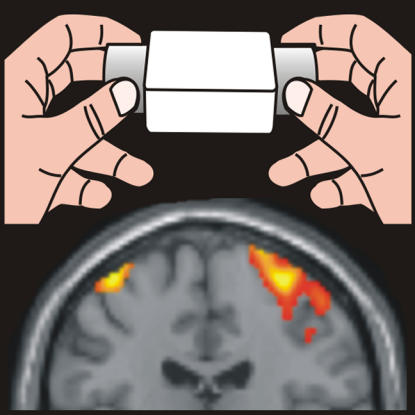
When the rules of a bimanual task changed, the center of activity switched between hemispheres in motor-control areas in the brain's cortex and cerebellum.

